# Macroporous Polyimide Aerogels: A Comparison between Powder Microparticles Synthesized via Wet Gel Grinding and Emulsion Processes

**DOI:** 10.1021/acs.langmuir.2c02696

**Published:** 2023-01-27

**Authors:** Shima Dayarian, Hojat Majedi Far, Liu Yang

**Affiliations:** † Department of Mechanical and Aerospace Engineering, University of Strathclyde, 75 Montrose Street, GlasgowG1 1XJ, United Kingdom; ‡ Blueshift Materials Inc., Spencer, Massachusetts01562, United States

## Abstract

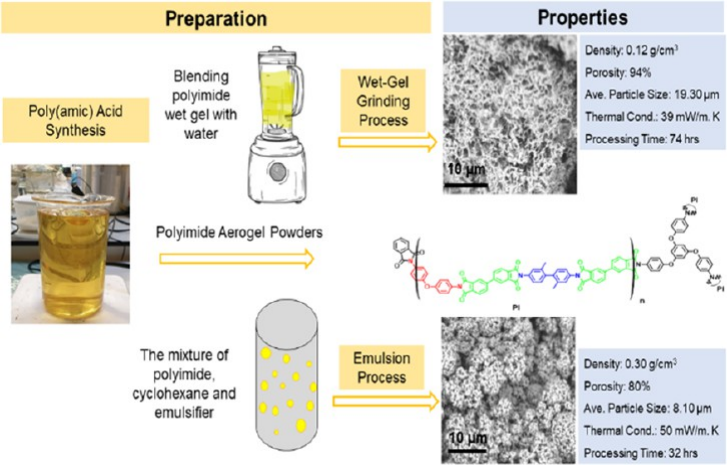

It is noteworthy to mention that synthesizing the polyimide aerogel powder, which is carried out in this study, benefits from two advantages: (i) the powder particles can be used for some specific applications where the monolith is not suitable and (ii) there is a possibility to investigate how a polyimide aerogel monolith can be made through the polyimide powder to reduce its cost and cycle time. In this study, two straightforward methods, wet gel grinding and emulsion, are introduced to prepare polyimide aerogel powders using ambient pressure drying. The microscopic properties of interest, including skeletal and porous structures, microparticle size and assembly, combined with macroscopic properties such as thermal stabilities and conductivities (0.039 W/m·K), confirm that the fabricated microparticles with a size in the range of 7–20 μm and porosity in the range of 65–85% are thermally stable up to 500 °C.

## Introduction

Aerogels are solid materials with the lowest density and various beneficial properties that make them suitable for a broad range of applications. Polyimide (PI) aerogels have attracted an exceptionally high level of attention due to their highly porous structures, low density, high thermal stability, excellent mechanical properties, low dielectric contents, and low thermal conductivity.^1−3^ These properties render PI aerogels ideal candidates for thermal insulation under vigorous conditions.^4,5^ Even if the fabrication process for PI is expensive and needs a tedious process compared to silica aerogel, these unique properties, combined with high absorption ability in recycling and filtration, make PI incredible to be used in different applications.^6^

Polyimide aerogels are synthesized by mixing dianhydrides with amines^7−9^ or isocyanates^10−12^ in the form of monolith or film.^13,14^ Recently, strong evidence has emerged that reducing the dimensions of the materials from a few millimeters to a micrometer or even a few nanometers gives rise to many unique properties in these materials. For example, the PI aerogel microparticles with a few micrometer sizes demonstrate a triple mass diffusion rate compared to monolithic varieties with similar porosities and surface areas. Therefore, there are a variety of applications in which such monoliths are unsuitable for use, including those requiring powders or microparticles such as catalysts, energy storage devices, drug delivery systems, scaffolds, or absorbers of small organic molecules.^15^

Powder particles can be made using a variety of techniques, including spray drying,^16−18^ an emulsion process,^19−23^ jet-cutting,^24−26^ and dry milling methods in which the powder is obtained using mechanical means such as milling or crushing of previously formed monolithic aerogel structures.^27,28^ However, irrespective of how they are made, these types of powders have been shown to exhibit weak mechanical properties.^15^ Concerning this, Lee et al. detailed the process of using swelling methods to manufacture polyimide aerogel spherical formation. Monomers such as pyromellitic dianhydride, 3,3′,4,4′-benzophenonetetracarboxylic dianhydride, and 4,4′-oxydiphthalic anhydrides were used to investigate their effect on the size of the produced pores. Unlike other types of methods for synthesizing the polyimide aerogel, cross-linkers and additives were not used. In addition, in this method, the spherical form is performed by self-assembly without using solvent exchange and supercritical drying. The pore size and pore volume of the produced particles were increased from 4 to 20 nm and 1.29 to 2.06 cm^3^/g simultaneously as a result of the surface area increasing from 54 to 88 m^2^/g.^29^

The literature reports two main methods for synthesizing aerogel microparticles. In the first method, splitting is applied to transform the aqueous polymer solution into microparticles, followed by the freeze-drying method.^30^ This method helps synthesize cellulose aerogel microparticles.^31^ In the second method, the aerogel microparticles are fabricated with the emulsion polymerization of precursor sol droplets stabilized by surfactants in an immiscible continuous liquid medium. The size of the particles for this method depends on the mixing speed, surfactant concentration, and dispersed phase content.^22^ The aerogel microparticles are obtained in the final stage after drying with a supercritical process.^32,33^ Silica aerogel microparticles can be derived from this method.

The oil-in-oil emulsion method was introduced by Teo et al. to produce micrometer-size voids in conjunction with inherently produced meso- and macropores in the gel form. They presented a range of 30–80 μm particle size for the produced PI microparticle aerogel by changing the concentration of F127 as a surfactant.^19^ Meanwhile, Ji et al. reported an oil-in-water-in-oil (O/W/O) multiple emulsion for preparing polyimide microsphere particles using Op10 and span-80 as emulsifiers.^23^ Commenting on their oil-in-oil emulsion process, Gu et al. demonstrated a reduction in the degree of shrinkage for the microparticles compared to monolithic using the oil-in-oil emulsion process.^15^ Their produced spherical microparticle with a 25 μm size and 80% porosity was synthesized with a fast solvent exchange method, which caused a reduction in the total shrinkage. In all of this literature, the process will be ended with supercritical drying. The equipment needed to conduct supercritical drying is costly and has associated running expenses for power, pressurized gasses, and maintenance. In addition, using supercritical drying, there is a limitation in terms of the size and type of the sample. However, ambient pressure drying can be used for all materials, such as plastics and certain metal alloys, which cannot survive in supercritical conditions in different forms and sizes.^34−36^

**Scheme 1:**
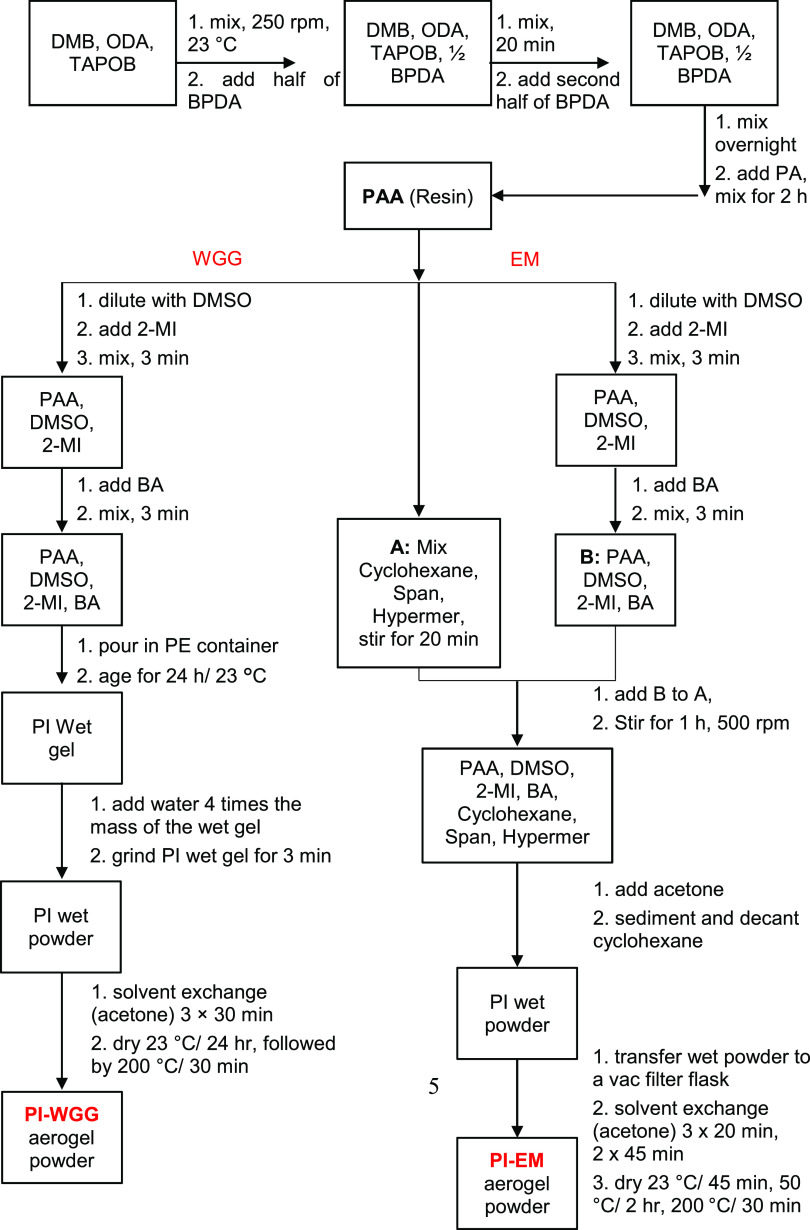
Schematic Representation of WGG and EM Synthetic Processes Used in This Study for the Preparation of PI Aerogel Microparticles PAA: polyamic acid (resin); WGG: wet gel grinding; EM: emulsion; PI-WGG: polyimide aerogel powders by WGG; PI-EM: polyimide aerogel powders by EM.

The present study was conducted to detail the production of polyimide aerogel powders with controlled particle sizes using wet gel grinding (WGG) and oil-in-oil emulsion (EM) methods with a short solvent exchange process. The solvent exchange can be completed with acetone using these techniques in less than 3 h. For both methods, first, polyamic acid (PAA) was synthesized by mixing 4,4′-oxydianiline (ODA) and 4,4′-diamino-2,2′-dimethylbiphenyl (DMB) as a diamine and 3,3′,4,4′-biphenyltetracarboxylic dianhydride (BPDA) as an anhydride in two additions. 1,3,5-Tris(4-aminophenoxy) benzene (TAPOB) also was added as a branching agent in this step.^37^ 2-Methyl imidazole (2-MI) as the catalyst and benzoic anhydride (BA) as the dehydrating agent were used to complete the imidization process. Finally, low-density, highly porous aerogel powders were obtained after solvent exchange and drying under ambient pressure. All of the steps involved in these techniques are summarized in Scheme 1.

## Experimental Section

### Materials

TAPOB was purchased from Wakayama Seika Kogyo. DMB was purchased from TCI. Dimethyl sulfoxide (DMSO, ≥99% Reagent Plus), ODA, BPDA, phthalic anhydride (PA), BA, 2-MI (99%), acetone (technical grade), and cyclohexane (≥99% ACS reagent) were purchased from Sigma-Aldrich. Hypermer 1599A and Span 85 were purchased from Croda. All reagents and solvents were used without further purification.

### Synthesis of Polyamic Acid

All formulations for the synthesis of PAA, PI-WGG, and PI-EM aerogel powders are listed in Appendix I.

At first, PAA was synthesized as follows: 225.81 g of DMSO was added to a precleaned baffled reactor. Then, 4.98 g of DMB, 4.69 g of ODA, and 0.30 g of TAPOB were added simultaneously and stirred with the solvent at room temperature until all components were fully dissolved. Every 20 min, 6.71 g of BPDA was added twice to the mixture and allowed to dissolve completely after each addition. The resulting homogeneous mixture was left to stir overnight. The next day, 0.82 g of PA was added to the mixture and stirred for 2 h. The resulting pale orange solution PAA was drained from the reactor, weighed, and ready for use in the synthesis of PI aerogel powders in the following sections (see Figure S1).

### Synthesis of PI-WGG Aerogel Powders

In a 1 L glass beaker, 250.00 g of PAA was diluted by adding DMSO in  ratios of 0.0, 0.5, 1.0, and 1.5 and the sol was stirred for 3 min using a mechanical stirrer. 2-MI (17.25 g) was added, stirring the PAA for 3 min. BA (52.25 g) was added to the sol, and the mixture was stirred for a further 3 min. The sol was then transferred to a polyethylene container for the gelation, which took about 30 min. After 24 h of aging under ambient conditions, the resulting PI wet gel was ground using a blender for 3 min. For grinding, water was used 4 times the volume of wet gel to prevent the explosion during the blending process. The wet powder was solvent-exchanged/vacuum-filtered with acetone 3 times every 30 min. Finally, PI-WGG aerogel powders were obtained after drying at room temperature for 24 h and 30 min at 200 °C (see Figure S2).

During the mechanical milling process on the porous materials, the structure will be broken down through its weak points related to its pores. Therefore, some original pores are expected to be destroyed during grinding. However, the pores smaller than the size of the particle may survive. In this work, the stress applied to the gel is significantly reduced due to the grinding on the wet gel, which helps maintain the porous structure within the gel particles.

### Synthesis of PI-EM Aerogel Powders

In a 1 L glass beaker, emulsifiers, 9.50 g of Span 85, and 3.25 g of Hypermer 1599 were added to 389.50 mL of cyclohexane, and the solution was stirred using a mechanical stirrer until emulsifiers were fully dissolved (∼20 min). These emulsifier agents reduce the surface tension between the two dissimilar liquids.^38^ It has been found that a combination of emulsifier with different behavior (one is hydrophilic, whereas the other is more hydrophobic) produces a better emulsion than a single emulsifier with the intermediate HLB number.^39^ Therefore, in this work, mixed surfactants were used. In a separate sealed round-bottom flask, 250.00 g of PAA was diluted by adding DMSO in  ratios of 0.0, 0.5, 1.0, and 1.5 and the sol was stirred for 3 min. Then, 17.25 g of 2-MI and 52.25 g of BA were added stepwise by mixing for 3 min between each addition. Subsequently, the mixture of PAA, DMSO, and catalyst was added to the solution of emulsifiers in cyclohexane slowly using a pipet. The mixture was stirred for 1 h with a stirring rate of 500 rpm. Then, the cyclohexane was decanted, and the DMSO layer was poured into a beaker containing 500 mL of acetone. After 15 min, the PI-EM wet powders were collected in a vacuum filtration funnel. Finally, PI-EM aerogel powders were spread on a large enough tray and air-dried for 45 min at room temperature, followed by 2 h at 50 °C and 30 min at 200 °C (see Figure S3).

**Scheme 2:**
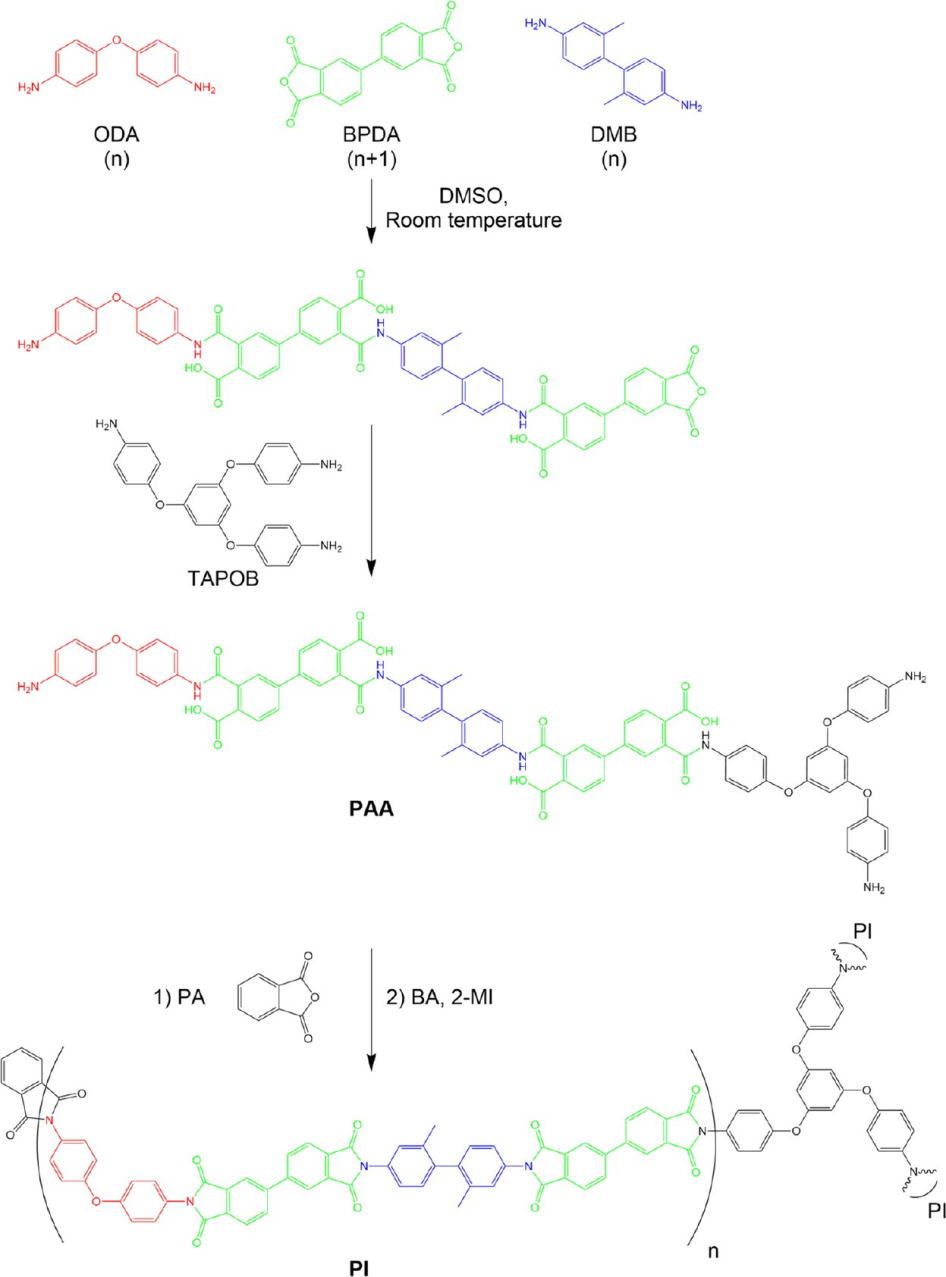
Synthesis of PAA and PI Aerogel from Amines (DMB, ODA, TAPOB) and Anhydrides (BPDA, PA) and Catalyzed with 2-MI

The chemical reaction and the structure of PI aerogels produced in this study are shown in Scheme 2. Samples obtained using WGG are referred to as PI-WGG-xx, and samples prepared using EM are referred to as PI-EM-xx, with the suffix “-xx” denoting the ratio of DMSO (g) to resin (g) in the preparation of the powders.

### Characterization Methods

Scanning electron microscopic (SEM) images were taken using a PHENOM Pro from NanoScience instruments. Image-Pro analysis was applied to the optical images to measure the particle sizes. The microscopic images obtained by Olympus optical microscope were opened in Image-Pro. Using the manual split, the particles that overlapped were split and introduced as individual particles into the software. To get the microscopic images, the powder is poured onto a small glass plate, and by shaking the plate smoothly, the particles are dispersed separately on the plate as much as possible. The particle in this work is defined as the smallest object that remains on the glass plate after shaking the plate to get the microscopic images. Figure S4 shows different possible diameters for a nonuniform particle. In our measurement, the size of the particle is defined as the average of diameters in different directions for each particle. For each powder type, at least 500 particles were determined using different microscopic images, and their size was measured using the Image-Pro analysis.

In addition, for measuring the small particles, the following equation was used^40^
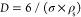
1

In which σ is the BET surface area, ρ_s_ is the skeletal density, and *D* is the diameter of the particle.

Brunauer–Emmett–Teller (BET) specific surface areas and mesoporosity of powders were determined with nitrogen sorption porosimetry at 77 K using an ASAP2420 from Micromeritics Instrument Corp. Before the analysis, samples were degassed for 30 min at 50 °C, followed by 120 min at 120 °C under a vacuum at 10 mmHg.

Complete pore size distributions and bulk densities were measured with low- and high-pressure sweep mercury intrusion porosimetry (MIP) using an Autopore V model 9605.^41^ A sweep of 0–30 psi, followed by a sweep of 30–33000 psi, was applied for low and high pressures, respectively. The % porosities were calculated using^42^
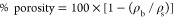
2where ρ_b_ is the bulk density (g/cm^3^) using MIP, and ρ_s_ is the skeletal density (g/cm^3^) measured on a Micromeritics Accupyc II 340 helium pycnometer.

Thermogravimetric analysis (TGA) was conducted to compare the amount of residual solvent and thermal stability in the powders using a TA Instruments Q50 thermogravimetric analyzer. The % residual solvents (RS) were measured using the % weight loss at 200 °C under nitrogen.

To optimize the solvent exchange and drying steps to obtain the powders with the lowest RS, the time interval between the washing and drying steps was varied as well as the number of washes; then, the RS was measured using TGA. The best condition that gave the lowest RS in PI-WGG powders was “solvent exchanging (acetone) 3 times every 30 min, then drying for 24 h/23 °C, followed by 30 min at 200 °C” and in the case of PI-EM powders was “solvent exchanging (acetone) 3 times every 20 min and 2 times every 45 min, then drying for 45 min at 23 °C, 2 h at 50 °C, and finally 30 min at 200 °C”. Representative data for the emulsion process are shown in Table S4.

For thermal stability and degradation studies, the temperature was increased from 20 to 700 °C in air at a heating rate of 10 °C/min.

Thermal conductivities were measured using a XIATECH TC3000 thermal conductivity meter by hot wire according to ASTM C1113.^43^ A sample holder with a volume of 110 cm^3^ and a thickness of 4.5 cm was filled with the powders.

## Results and Discussion

**Figure 1:**
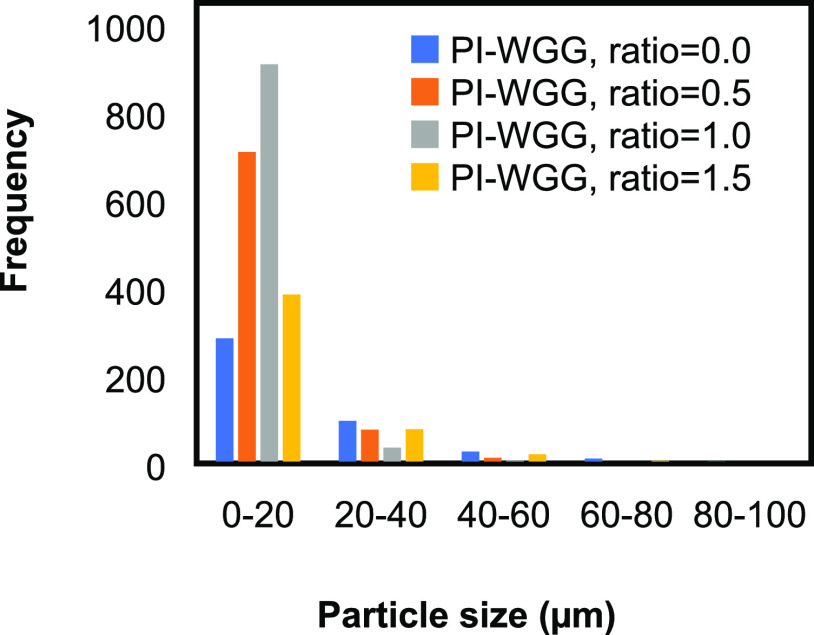
Particle size distribution for PI-WGG powders at different dilution ratios.

**Figure 2:**
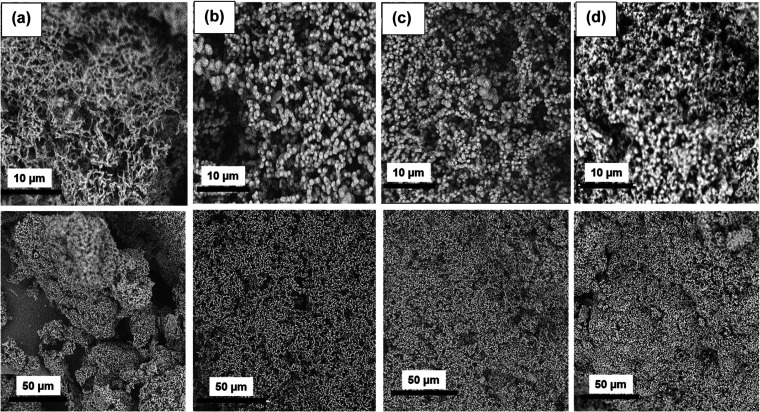
SEM for PI-WGG powders with DMSO/PAA ratios of (a) 0.0, (b) 0.5, (c) 1.0, and (d) 1.5.

The distribution of the measured diameters for wet gel ground powders is shown in Figure 1. It can be observed that for all of the ratios, a narrow distribution is observed in the range of 3–20 μm. Looking closely at the SEM images in Figure 2 for each ratio, it can be perceived that the particles measured in this work consist of different primary particles, which connect to form the larger agglomerates. The agglomerations can develop during the blending process, solvent exchange, and drying process.^42^ Considering the SEM image, the size of the particles becomes smaller by increasing the dilution. The surface of the wet gel ground particles is smooth; therefore, the porosity is mainly between those particles. Using the SEM images, it can be observed that dilution can change the morphology of the particles. On the other hand, the fibrillar network can be seen for the PI-WGG particles in a different ratio. This could be due to the gelation occurring in the two-dimensional (2-D) layer and then growing in the three-dimensional (3-D) network inside the particles.

**Figure 3:**
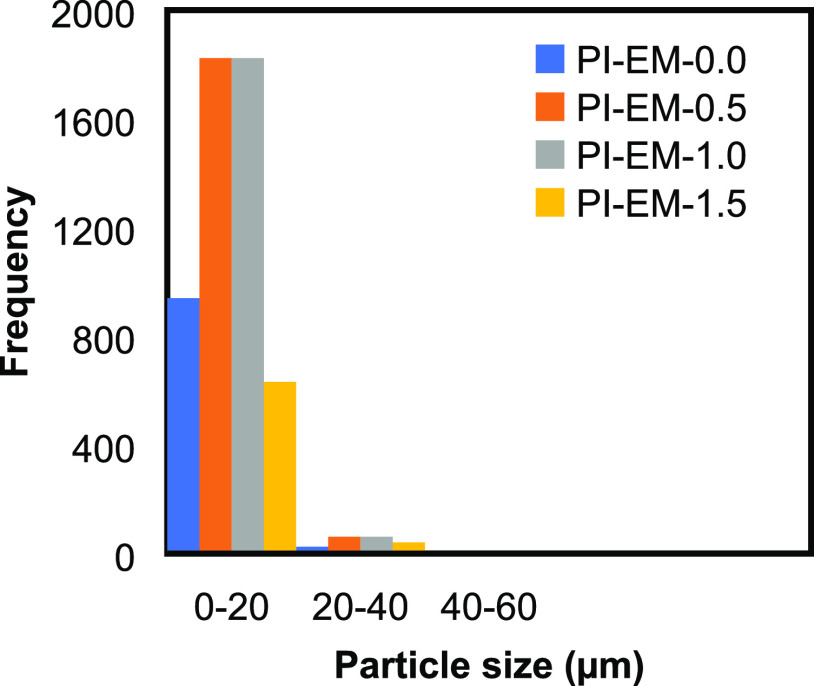
Particle size distribution for PI-EM powders at different dilution ratios.

**Figure 4:**
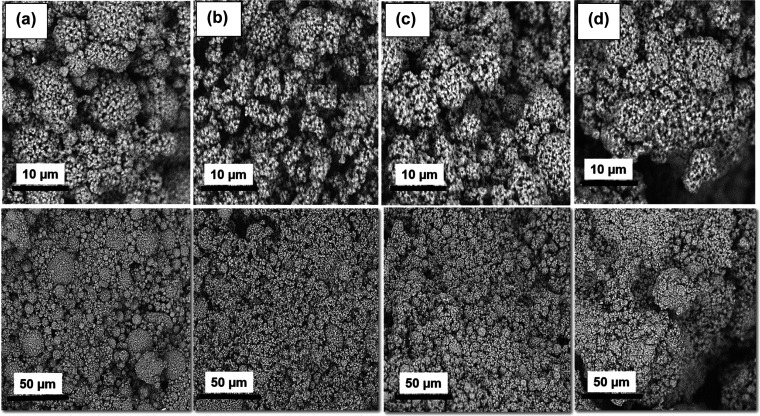
SEM images for PI-EM powders with DMSO/PAA ratios of (a) 0.0, (b) 0.5, (c) 1.0, and (d) 1.5.

The graph of the particle size distribution for the PI-EM powders is presented in Figure 3. The right-skewed distribution can be observed for all of the ratios. As it is clear, wider distributions are formed for PI-EM samples compared to those for the PI-WGG, which generally indicates the less uniform particles in those samples. A broad particle size distribution typically occurs for the particles made using the emulsion method. This is due to a wide shear rate during the mechanical mixing and agglomeration of the particles, which is caused by coalescence.^22^ For example, the microparticles synthesized by Gu et al. using the oil-in-oil emulsion process were 10–90 μm.^15^ The average particle size is calculated using the different measured particles with image-Pro and is presented in Table 1. By increasing the dilution in the system and reducing the amount of polymer in the manufacturing process, smaller particles were formed due to increasing the repulsive force (see Figure 4).

**Table 1:** Properties of PI-WGG and PI-EM Aerogel Powders

sample DMSO (g)/PAA (g)	MIP surface area σ (m^2^/g)	N_2_ sorption, average pore diameter (nm)^a^	MIP average pore diameter (nm)	MIP bulk density ρ_b_(g/cm^3^)	skeletal density ρ_s_(g/cm^3^)^b^	porosity (%)^c^	smallest particle size (μm)^d^	average particle size (μm)^e^	residual solvent (%)^f^	10% weight loss (°C)^g^	thermal conductivity (W/m·K)^h^
PI-WGG											
0.0	16.90	37.20 ± 0.10	1743	0.12	1.78 ± 0.003	94	0.20	19.30	0.59	518	0.039 ± 0.001
0.5	9.01	36.80 ± 0.30	1222	0.28	1.54 ± 0.001	82	0.43	11.91	0.16	536	0.044 ± 0.003
1.0	13.50	24.80 ± 0.81	756	0.29	1.52 ± 0.001	80	0.29	7.82	0.44	539	0.052 ± 0.002
1.5	34.70	23.31 ± 0.04	147	0.48	1.38 ± 0.003	65	0.13	14.21	0.57	543	0.064 ± 0.004
PI-EM											
0.0	7.60	38.50 ± 0.11	1046	0.34	1.37 ± 0.005	75	0.58	6.80	0.95	537	0.061 ± 0.006
0.5	18.40	37.70 ± 0.10	505	0.32	1.41 ± 0.007	78	0.23	7.93	0.68	521	0.052 ± 0.002
1.0	32.60	33.21 ± 0.03	240	0.30	1.39 ± 0.007	80	0.13	8.12	0.78	526	0.050 ± 0.003
1.5	43.50	25.60 ± 0.04	296	0.24	1.40 ± 0.006	84	0.10	16.33	0.83	525	0.068 ± 0.005

a Average of three samples; single point at *V*_max_.

b Single sample, 50 measurements.

c Via 100 × [1 – (ρ_b_/ρ_s_)].

d Via 6/(σ × ρ_s_).

e Average of more than 500 particles (Image-Pro) from optical images.

f Using TGA under nitrogen: % residual solvent = 100% – % wt loss (200 °C); see Table S4 of the appendix for the best washing and drying conditions that resulted in the lowest % residual solvents.

g Using TGA in air.

h Average of three measurements.

However, on the other hand, for the EM process, the high-speed mixing applied in the process has caused agglomeration between the small particles, and therefore, the average size of the particle has increased. The particle size distributions are in line with the density correlation. For powder with a ratio of 1.5, there is a wider distribution compared to other ratios with more particles in the bigger particle size. These results show that the presence of small particles in the ratio of 0.0–1.0 causes a better placement for the particles to sit close to each other, making them denser. In the case of PI-EM-1.5, higher porosity and lower bulk density are observed due to the presence of larger particles. That is also why the pore size is large in this sample.

The surface morphologies of the EM powders were examined using SEM, and the results are shown in Figure 4. The micrometer-sized spherical particles are observed for PI-EM samples with 0.10–0.58 μm sizes, as calculated using eq 1. The addition of DMSO created agglomerated particles in the PI-EM powders, which made the particles larger by increasing the level of DMSO. The soft and open pore’s structure can be observed for the emulsion particles in the SEM image. The smooth surfaces in the synthesized particles caused possible deformation during the mixing and washing. Gu et al. achieved the same result. They reported no regular spherical shape for PI aerogel particles.^15^ The hollow microspherical particles with particle size in the range of 295.5–1479.6 μm are introduced by Luo et al. Even though they believed in reporting a method for synthesizing the PI particles with controlled particle size using the smashing process, the particles’ fabricated pores and mechanical properties will be affected.^44^

**Figure 5:**
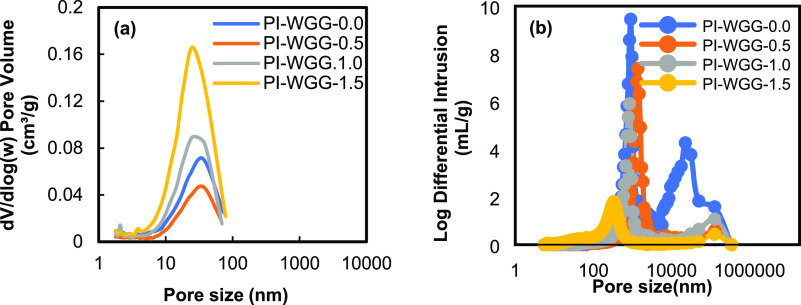
Pore size distributions of PI-WGG powders were measured using (a) N_2_ sorption and (b) MIP.

**Figure 6:**
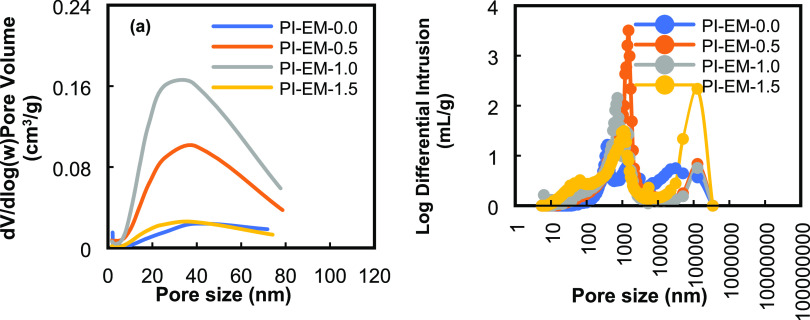
Pore size distributions of PI-EM powders were measured by (a) N_2_ sorption and (b) MIP.

Figures 5 and 6 show the pore size distribution for PI-WGG and PI-EM powders at different ratios using the N_2_ sorption and MIP. The desorption branch of the isotherms was used to calculate the Barrett–Joyner–Halenda (BJH) pore size distribution.^45^ The average mesopore sizes for both types of powder are presented in Table 1 and show the exact change by increasing the DMSO level ranging from 23 to 38 nm. The presence of a low rate in the quantity of adsorbed gas at a low relative pressure of up to 0.6 plus a sharp increase in this correlation at a high relative pressure (*P*/*P*_0_ = 0.9) confirms that most of the pores are mesopores and macropores with relatively lower mesopore content (see Figure S5). Gu et al. show a short saturation plateau for the PI aerogel microparticle using the O/O emulsion method.^15^ The nitrogen adsorption isotherms for both types of powder increase above *P*/*P*_0_ = 0.9 but do not reach the saturation plateau, indicating that they are type II isotherms. The calculated total pore volume for PI-WGG and PI-EM using the quantity of the adsorbed gas at *P*/*P*_0_ = 0.9 shows that the pore volume is in the range of 0.043–0.123 and 0.022–0.142 cm^3^/g, respectively. Considering the area under the pore size distribution plots, which is approximately equal to the cumulative pore volume, PI-WGG-1.5 and PI-EM-1.0 have the highest amount of pore volume. This conclusion is consistent with the observation on the N_2_ isotherm plot because the wider hysteresis loop can be observed for these two samples compared to that of other samples in their groups. The reported surface area in this work (7.60–43.5 m^2^/g) shows a relatively low value compared to what was reported in the literature, which is caused by the ambient pressure drying method.^46,47^

The MIP for all PI-WGG and PI-EM samples represents the pores in the range of 2–250 μm and bimodal distributions, indicating that the pores are mostly mesopores and macropores. A narrow peak can be observed for all conditions and inter- and intrapore distributions. Same as what was observed in gas adsorption results, the same behavior is observed in the average pore sizes obtained from MIP; as for PI-WGG, by increasing the dilution from 0.0 to 1.5, the MIP average pore size changes from 1743 to 147 μm and as a result for PI-WGG-1.5 by increasing the pressure up to 300 Psia, the volume of the pores, which are filled with mercury, is less than 1 cm^3^/g (see Figure S6). The MIP pore size distribution for PI-WGG samples shows that by increasing the dilution and, therefore, the presence of a smaller particle, a reduction will happen in the volume of the pores.

For PI-EM-0.0 in Figure S6b, due to the presence of larger pores (1046 μm), a large volume of the pores was filled with mercury by adding very low pressure at the beginning. However, by increasing the dilution as discussed, the average size of the pores becomes smaller; therefore, higher pressure must be applied to fill the pores.

From these measurements, it can be concluded that for investigating the pore structure and size of the samples, both N_2_ sorption and mercury intrusion need to be used because nitrogen rapidly infiltrates small pores in the interior of microparticles. However, mercury cannot infiltrate into the interior of the pore network, which is smaller than the surface pores. Therefore, small pores in the interior of microparticles are measured by gas adsorption and pores on the surface of the particles and between the particles are measured by MIP.^48^

**Figure 7:**
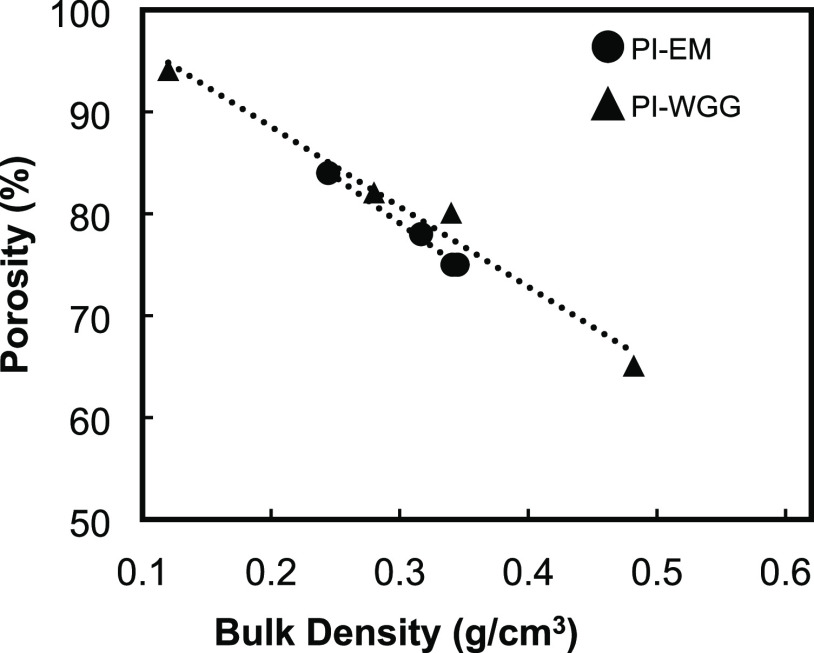
Porosity–density profiles for PI-WGG and PI-EM powders.

A linear correlation between % porosities and bulk densities for all samples can be seen in Figure 7. A 30% reduction in the porosity of the PI-WGG samples can be observed by increasing the density from 0.12 g/cm^3^ in PI-WGG-0.0 to 0.48 g/cm^3^ in PI-WGG-1.5, which is caused by dilution (Figure 7). By increasing the dilution for PI-WGG particles, as was shown before, the size of the particles is decreased. Therefore, they pack more into each other, indicating an increase in the density and a reduction in the porosity. However, for PI-EM powders in Figure 7, porosities linearly show a 10% increase after bulk densities have dropped by 30% due to dilution. The most significant density belongs to PI-EM-0.0, 0.34 g/cm^3^, and 75% porosity, whereas PI-EM-1.5 has the lowest density of 0.24 g/cm^3^ and 84% porosity. The mixing and stirring speed can affect the particle size distribution, growth and amount of agglomeration, and nucleation. There is a moderate stirring speed for each process, which can improve the mixing process and create a more uniform environment. For example, Hussain et al. show that the size of the particle for chitosan nanoparticles is reduced gradually from 543 ± 32 to 167 ± 18 nm when the stirring speed is increased from 200 to 700 rpm. However, in the next increment from 800 to 1000 rpm, the average particle size is increased from 432 ± 34 to 712 ± 42 nm.^49^ These results agree with other published studies.^50^

**Figure 8:**
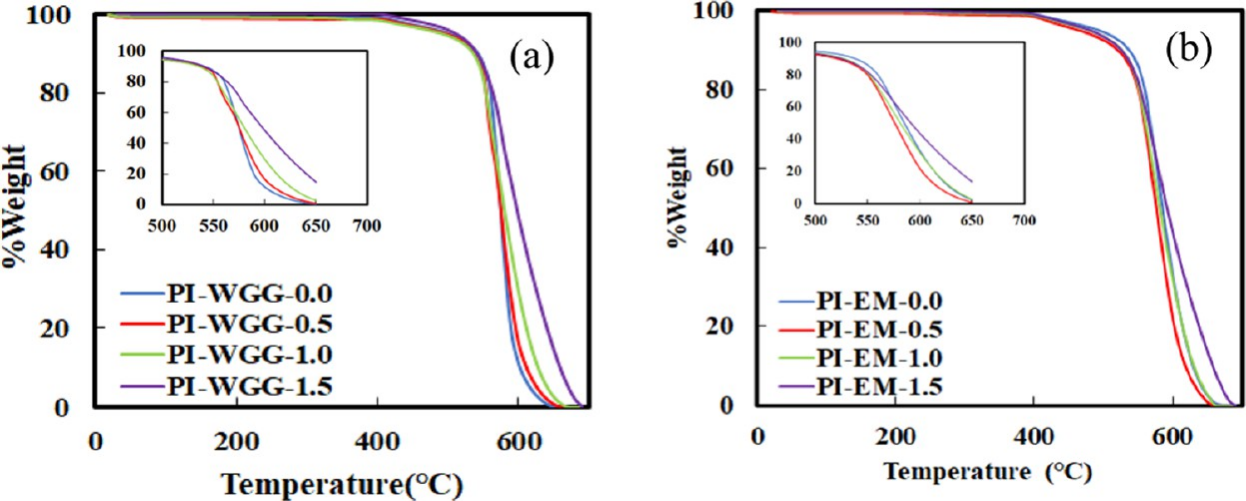
% Weight as a function of temperature for (a) PI-WGG and (b) PI-EM powders.

Figure 8 represents the weight change against the temperature for all powders. PI-WGG powders are thermally stable up to 430 °C. In the next step, for all of the ratios, they decompose at 540 °C and finally burn off at 700 °C. Table 1 shows that dilution in the wet gel ground particles causes an increase in the 10% decomposition temperature from 518 to 543 °C. Figure 8b presents the thermal behavior of PI-EM powders under increasing temperatures. The powders with different ratios are stable up to 410 °C, fully decomposing at 700 °C. The 10% weight losses of PI-EM powders take place at temperatures between 521 and 538 °C. As there is no significant weight loss before the decomposition point, it can be confirmed that the solvent exchange and drying process removed all of the solvents. The residual solvent for all of the powders was also measured by TGA and is presented in Table 1. For this measurement, the temperature was increased under air from 20 °C up to 200 °C with a heating rate of 10 °C/min and then continued as an isotherm step for 15 min. The residual solvents for PI-WGG and PI-EM powders are <0.59 and <0.95%, respectively. Those values, however, do not seem to correlate with the sample’s pore sizes.

**Figure 9:**
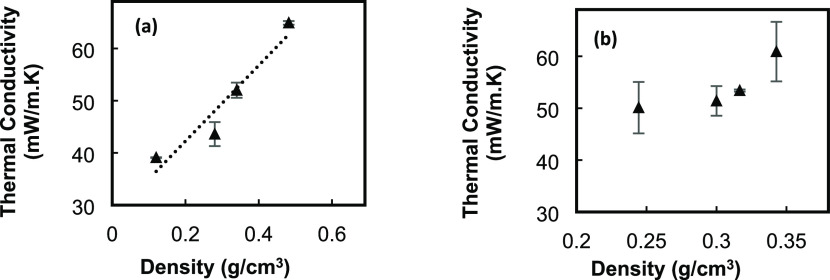
Thermal conductivity–density profiles for (a) PI-WGG and (b) PI-EM powders.

**Figure 10:**
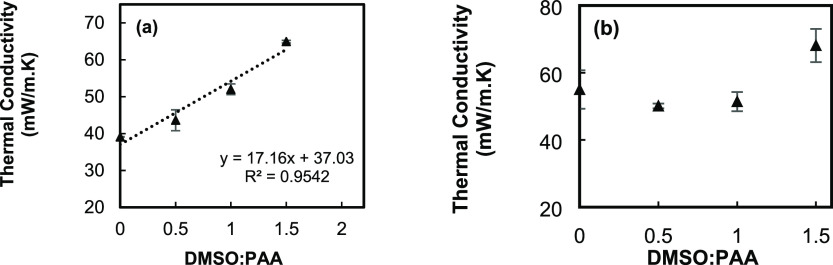
Correlation of thermal conductivity and dilution ratio for (a) PI-WGG and (b) PI-EM.

To measure the thermal conductivity using the A XIATECH TC3000E, the sensor with a 1 V heating voltage was placed between the top and bottom of samples in the sample holder. Figure S7 shows the equipment that is used for these measurements. The correlation of the thermal conductivity with density is shown in Figure 9. For both types of powder, thermal conductivity of the samples is increased by increasing the density of the powders. Figure 10 shows the correlation of thermal conductivity with the ratios for all types of powder. For wet gel ground particles, a linear correlation can be observed with a 40% increase in thermal conductivity by increasing the ratio from 0 to 1.5 (Figure 10a). Increasing the DMSO level in the wet gel ground method causes a reduction in the particle size. Smaller particles can get closer to each other and transfer the heat faster. For EM powder (Figure 10b), due to increasing the agglomeration of particles caused by the high speed of stirring, 500 rpm, the particle size becomes bigger, and the thermal conductivity is first decreased and then starts to increase in PI-EM-1.5 samples.

**Figure 11:**
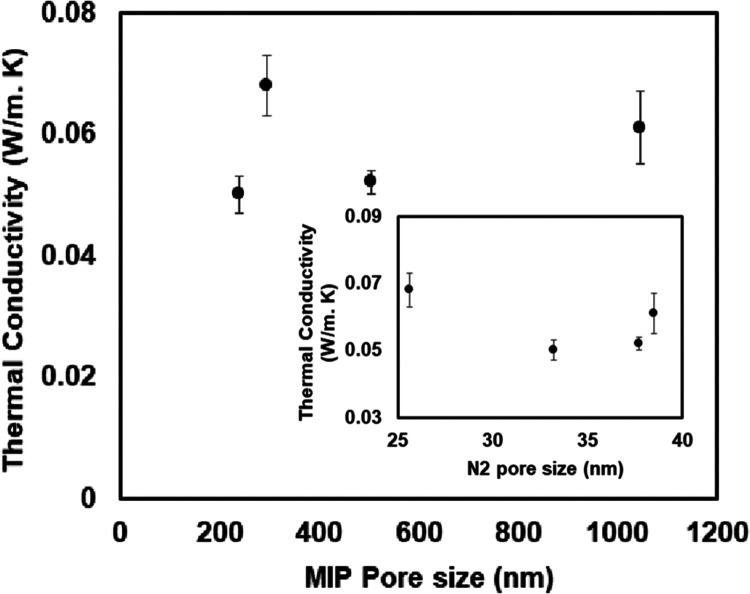
Correlation of thermal conductivity and pore size for PI-EM.

Figure 11 presents the correlation of thermal conductivity of the PI-EM particles and MIP and N_2_ sorption pore size. The MIP correlation shows an increment in the thermal conductivity for the larger pores, as is expected. However, the thermal conductivity is decreased for larger pores, which are detected by N_2_ sorption. These confirm that interporosity has the main role in conducting the heat.

## Conclusions

Reducing the size of the particles has a positive effect on most of the thermomechanical properties of powders. In addition, it can reduce the manufacturing cost for the aerogel materials by, for example, reducing the time of the solvent exchange. In this work, two new procedures, WGG and EM, were developed for manufacturing polyimide aerogel powders with various particle sizes using different solvent levels.

The introduced methods produced PI aerogel powders with a solvent exchange, as short as 3 h, and ambient pressure drying. The effect of dilution was investigated on different properties of the particles, such as thermal stability, pore texture, and particle size. In terms of particle size, the EM process produced smaller microspherical particles compared to WGG powders. The addition of DMSO created agglomerates in both methods. In WGG powders, the bulk densities were increased by 75% by diluting from 0.0 to 1.5, while in EM powders, the bulk densities had a 30% reduction by adding the diluting agent (DMSO). Both produced powders are thermally stable at above 520 °C and have highly porous structures, containing up to 94 and 84% air within their microparticles. These techniques provide a basis for manufacturing PI aerogel powders on a large scale where time and cost matter.

## Supporting Information Available

Additional experimental details, formulation data and methods, photographs of experimental work, and plots of N_2_ sorption isotherm and MIP cumulative intrusion versus pressure (PDF)

## Abbreviations

PI: polyimide
WGG: wet gel grinding
EM: emulsion
PAA: polyamic acid
PI-WGG: polyimide aerogel powders by WGG
PI-EM: polyimide aerogel powders by EM
ODA: 4,4′-oxydianiline
DMB: 4,4′-diamino-2,2′-dimethylbiphenyl
BPDA: 3,3′,4,4′-biphenyltetracarboxylic dianhydride
TAPOB: 1,3,5-Tris(4-aminophenoxy) benzene
DMSO: dimethyl sulfoxide
PA: phthalic anhydride
BA: benzoic anhydride
2-MI: 2-methyl imidazole
MIP: mercury intrusion porosimetry
SEM: scanning electron microscopy
TGA: thermal gravimetric analysis
