# Supplementary material for: Macroporous Polyimide Aerogels: A Comparison between Powder Microparticles Synthesized via Wet Gel Grinding and Emulsion Processes
Source: Langmuir. 2023 Jan 27;39(5):1804–14. doi: 10.1021/acs.langmuir.2c02696 (PMC9910053; doi:10.1021/acs.langmuir.2c02696)
Supplement: Supplementary file 1 — la2c02696_si_001.pdf [file la2c02696_si_001.pdf]

Supporting Information

Macroporous Polyimide Aerogels: A Comparison Between Powder Microparticles Synthesized via Wet Gel Grinding and Emulsion Processes

[1]Shima Dayarian, [2]Hojat Majedi Far, [1]Liu Yang*

[1]University of Strathclyde, Department of Mechanical and Aerospace Engineering, 75 Montrose Street, Glasgow, G1 1XJ, United Kingdom

[2]Blueshift Materials Inc., Spencer, Massachusetts 01562, United States

**Corresponding Autor:* s.dayarian@strath.ac.uk

Content: **Page**

Appendix I. S1

Appendix II. S4

Appendix I. Formulations of polyamic acid (PAA), wet gel grinding (WGG) and emulsion (EM) processes.

In this section, the formulation data for the fabrication of polyamic acid, and polyimide aerogel powders using the wet gel grinding and emulsion processes are presented. The formulation for the powder processes is presented for a dilution ratio of 1.5. Using these data, the calculations for other ratios can be done easily.

In addition, the amount of residual solvents using TGA in the powders in order to investigate the proper solvent exchange and drying method are presented for EM process.

Table S1. Formulations for the synthesis of **PAA** (resin)

monomer	weight (g)	weight (%)
DMSO	225.81	90.32
TAPOB	0.30	0.12
DMB	4.98	1.99
ODA	4.69	1.88
BPDA	13.41	5.37
PA	0.82	0.33
total	250.01	100.00
total solid	24.20	
% solid content	−	9.68

Table S2. Formulations for the preparation of **PI-WGG** aerogel powders

DMSO (g)/PAA (g) = 1.5		
Monomer	Weight (g)	Weight (%)
DMSO	375.00	54.00
PAA	250.00	36.00
BA	52.25	7.52
2-MI	17.25	2.48
Total	694.50	100.00
% Solid content	−	13.49
% Actual Yield [a]		86 ± 2

[a] % Actual yield = mass of dried powder/mass of solid monomers, an average of 3 experiments.

Table S3. Formulations for the preparation of **PI-EM** aerogel powders

DMSO (g)/PAA (g) = 1.5		
Monomer	Weight (g)	Weight (%)
DMSO	375.00	34.19
Span 85	9.50	0.87
Hypermer 1599-A	3.25	0.30
Cyclohexane	389.50	35.51
PAA	250.00	22.79
BA	52.25	4.76
2-MI	17.25	1.57
Total	1096.75	100.00
% Solid content	−	9.68
% Actual Yield [a]		77 ± 6

[a] % Actual yield = mass of dried powder/mass of solid monomers, an average of 3 experiments.

Table S4. Residual solvents (RS) using TGA for different solvent exchanges and drying conditions for **EM** (DMSO (g)/PAA (g) = 1.5) process as described

Sample	Condition 1	Condition 2	Condition 3	Condition 4	Condition 5	Condition 6	Condition 7
Total residual solvent (%) [a]	7.33	7.81	1.30	0.92 ± 0.11	0.83 ± 0.20	0.80 ± 0.04	0.83 ± 0.02

Solvent exchanges (aka washes) were carried out in acetone in all cases. A similar study was conducted for **WGG** process.

Condition 1: 3 washes every 20 min; 2 washes every 45 min; dried at 23 °C/ 45 min, 50 °C/ 8 hr.

Condition 2: 3 washes every 20 min; 2 washes every 45 min; dried at 23 °C/ 45 min, 50 °C/ 18 hr.

Condition 3: 3 washes every 20 min; 2 washes every 45 min; dried at 23 °C/ 45 min, 50 °C/ 2 hr, 200 °C/ 15 min.

Condition 4: 3 washes every 20 min; 2 washes every 45 min; dried at 23 °C/ 45 min, 50 °C/ 18 hr, 200 °C/ 15 min.

Condition 5: 3 washes every 20 min; 2 washes every 45 min; dried at 23 °C/ 45 min, 50 °C/ 2 hr, 200 °C/ 30 min.

Condition 6: 3 washes every 20 min; 2 washes every 45 min; dried at 23 °C/ 45 min, 50 °C/ 18 hr, 200 °C/ 30 min.

Condition 7: 3 washes every 20 min; 3 washes every 45 min; dried at 23 °C/ 45 min, 50 °C/ 2 hr , 200 °C/ 30 min.

[a] from TGA under air: 100% - % wt loss (200 °C) = % residual solvent.

Conditions 5, 6, and 7 showed the lowest % RS. By increasing the time (condition 6) and the number of washes (condition 7) % RS did not improve, therefore, condition 5 was chosen as the best one.

Appendix II. Pictures of PI-WGG and PI-EM aerogel powders and characterization methods.

In this section, the pictures from different steps of the processes involved in the preparation of polyimide aerogel powders, particle size measurements and thermal conductivity instrument are presented.

The gas adsorption isotherms for both types of powders at different ratios of dilution as well as the correlation of cumulative intrusion with pressure obtained with MIP are presented to complete the presented data in the paper.

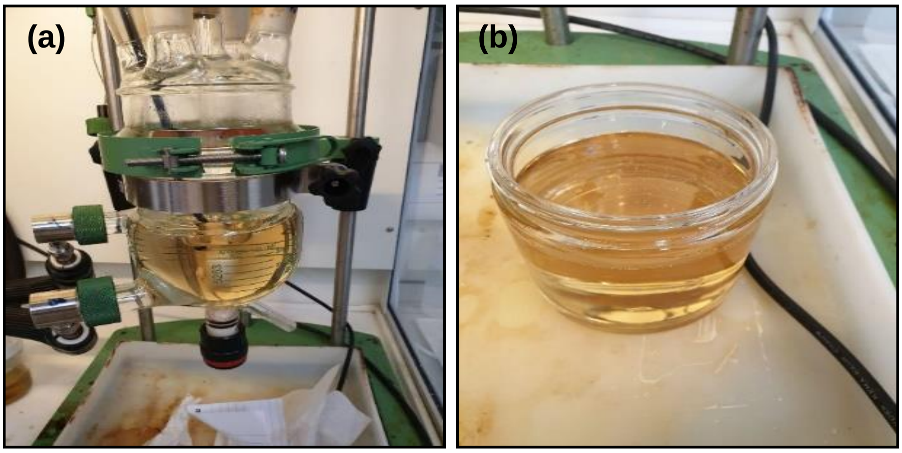

Figure S1. (a) The baffled reactor used for the synthesis of PAA; (b) PAA drained from the reactor after mixing the monomers overnight and addition of PA.

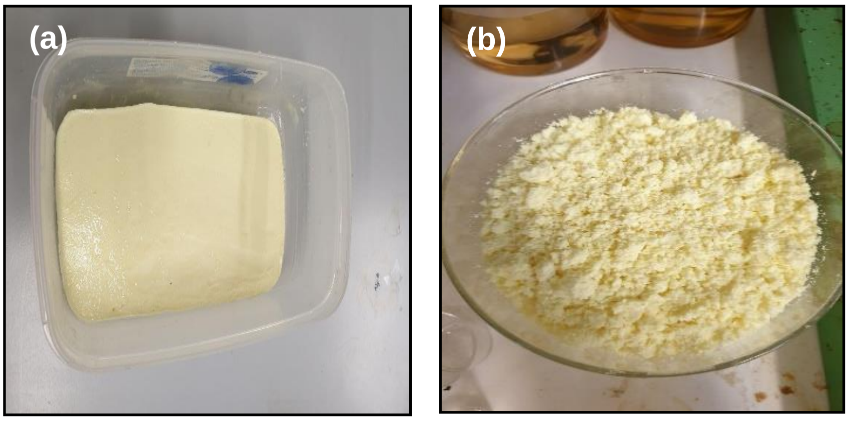

Figure S2. Preparation of PI-WGG aerogel powder: (a) PI wet gel after 24 hours of ageing at room temperature, (b) dried PI-WGG aerogel powder.

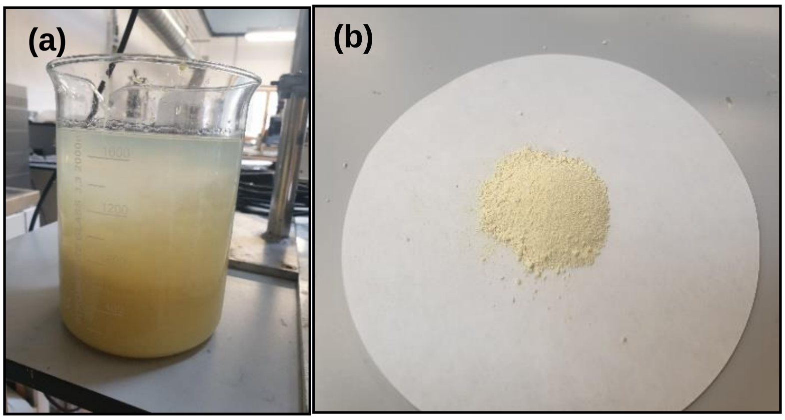

Figure S3. Preparation of PI-EM aerogel powder: (a) gelation and sedimentation of powders, (b) dried PI-EM aerogel powder.

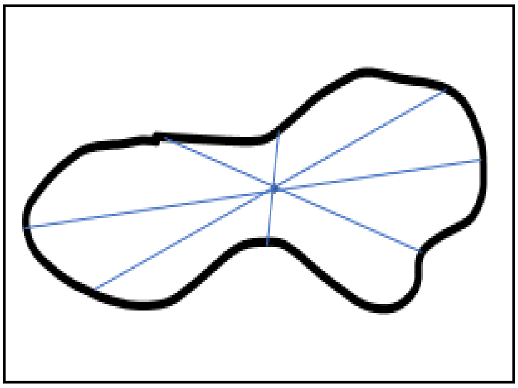

Figure S4. Possible diameters of a non-uniform particle accounted for the measurement of the particle sizes of powders.

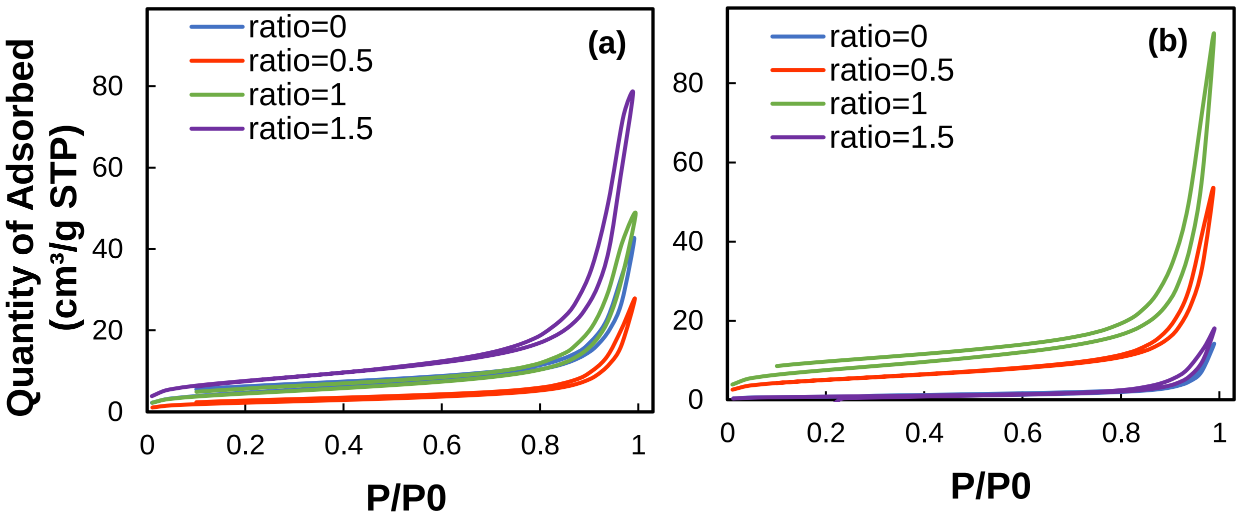

Figure S5. Nitrogen sorption isotherms at 77 K for (a) PI-WGG, and (b) PI-EM particles.

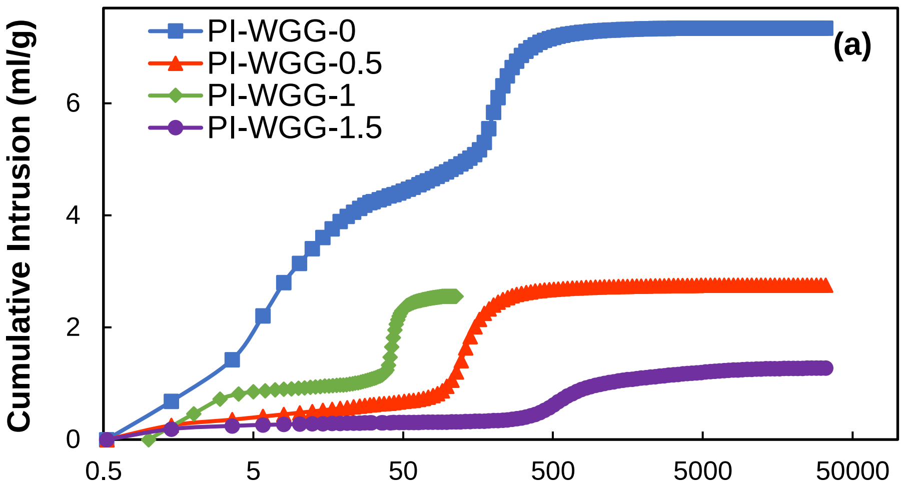

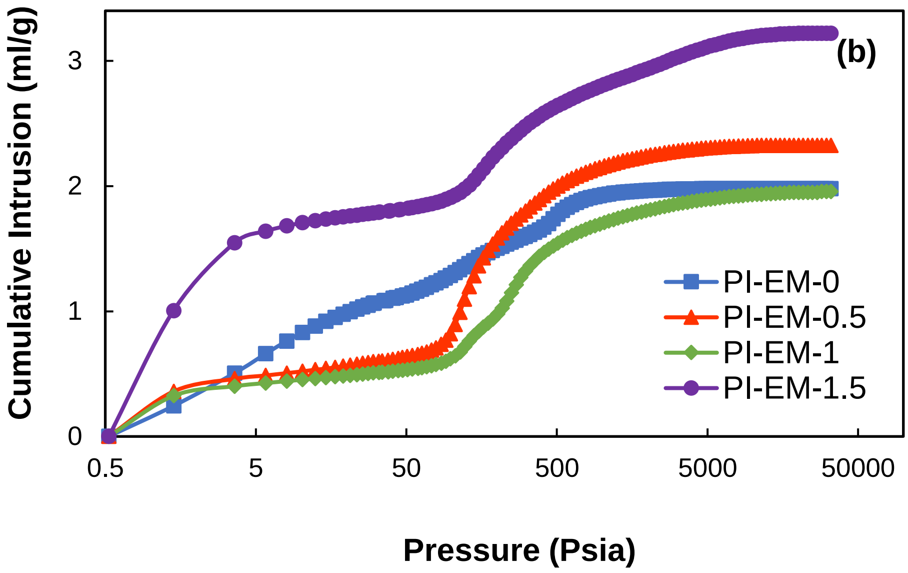

Figure S6. Cumulative intrusion versus pressure using MIP (a) PI-WGG, and (b) PI-EM particles.

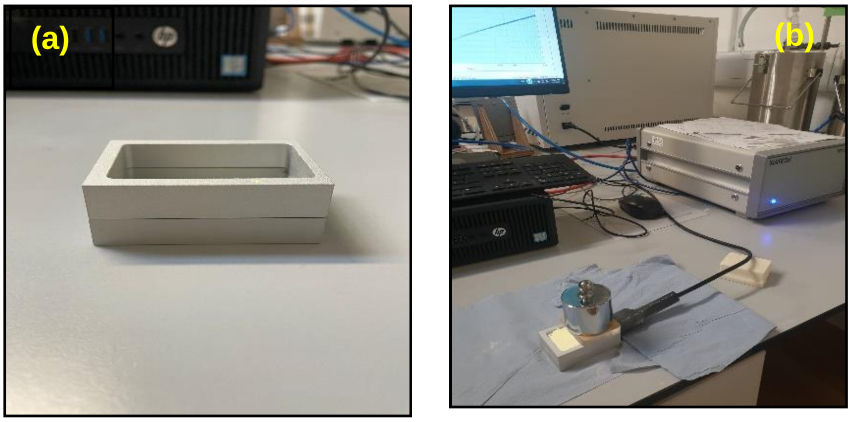

Figure S7. Apparatus for measuring the thermal conductivities of the powders: (a) sample holder, (b) XIATECH TC3000 hot-wire.
